# Do color similarity judgments vary with age, and do they reveal anything about qualia?

**DOI:** 10.1073/pnas.2521506122

**Published:** 2025-10-20

**Authors:** Jenny M. Bosten, Anna Franklin

**Affiliations:** ^a^Sussex Vision Lab, School of Psychology, University of Sussex, Falmer BN1 9QH, United Kingdom; ^b^Sussex Colour Group and Baby Lab, School of Psychology, University of Sussex, Falmer BN1 9QH, United Kingdom

In a new study that provides useful data for understanding the development of color appearance, Moriguchi et al. (1) applied multidimensional scaling (MDS) to color similarity judgments from a large sample of children and adults. They concluded that children have similar “qualia structures” to adults, with subtle differences providing evidence of developmental changes in color categories and in the distinction between warm and cool colors. Moriguchi et al. overlooked existing work on color similarity judgments in children and adults, undermining the originality of the approach. It is also unclear what, if anything, their results can reveal about color qualia. Moreover, we show here that the apparent changes in color similarity judgments with age can be explained by greater noise in their data from the younger age groups.

Moriguchi et al. did not place their study in the context of the large body of existing work that has applied MDS to color similarity judgments to investigate the geometrical structure of color appearance in adults (e.g., ref. 2) and children (3–5), and how it varies between individuals and groups (e.g., refs. 6 and 7). A convincing case was not made for why paired similarity judgments are most developmentally appropriate, especially given poor reliability from the youngest participants, and given that color appearance has been measured in children using other tasks (8, 9). What is new about Moriguchi et al.’s paper is not the approach but the framing: Where others have claimed to be investigating color representation or appearance, Moriguchi et al. claim to be investigating qualia structures. However, MDS solutions for color similarity ratings can be well accounted for by models of basic low-level color signals (e.g., ref. 10). Color is represented at many levels of the visual system in cone activities, in retinogeniculate pathways, and in distributed cortical mechanisms—developmental changes at any of these levels could affect perceived color similarity. Results from color similarity tasks cannot isolate processes specific to color qualia or their development.

**Fig. 1.**
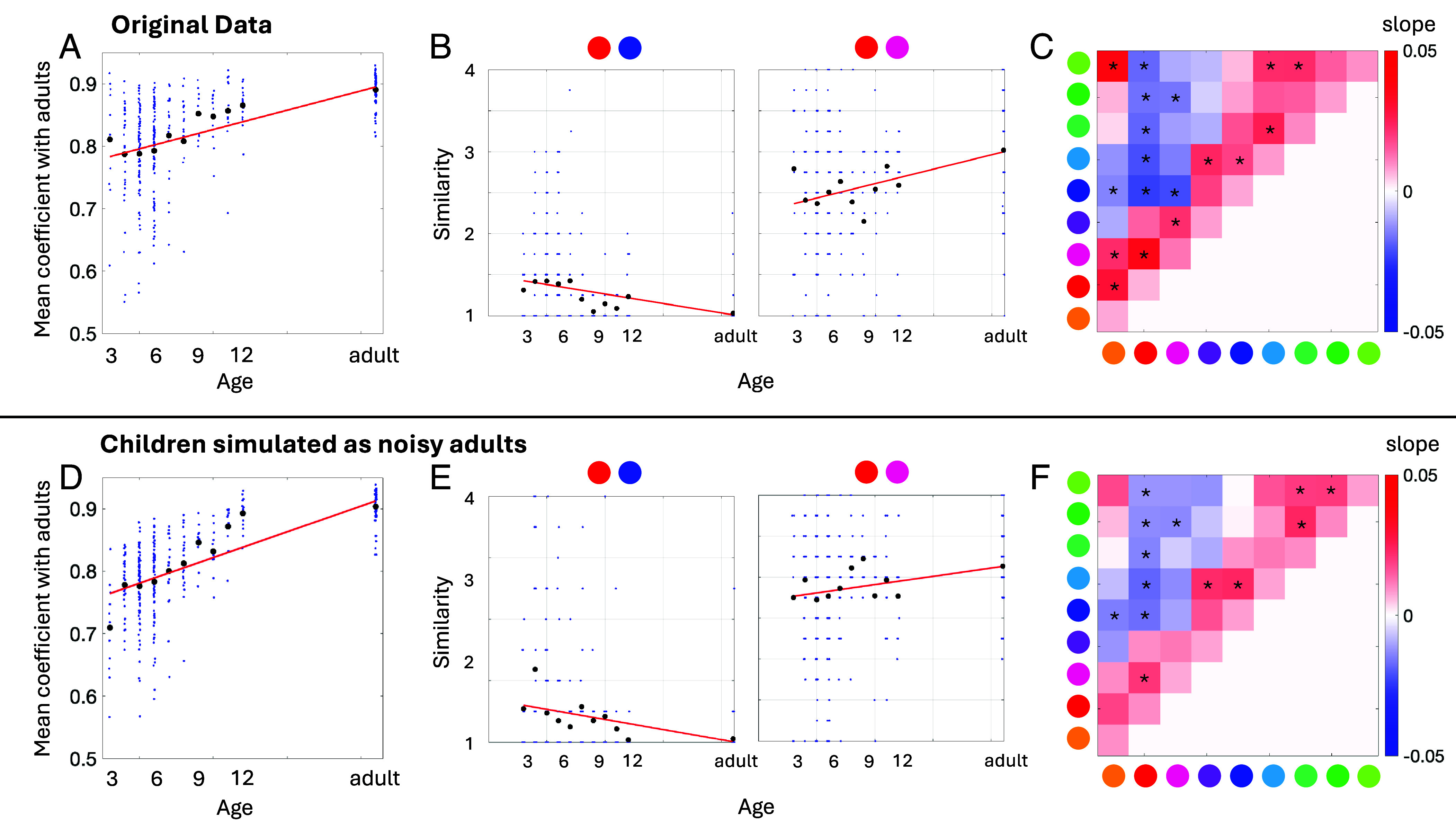
(*A*–*C*) Independent reproductions of Moriguchi et al.’s results, using data they made available. (*A*) Their figure 3B showing the correlation between similarity judgments for each participant and the adult group mean (blue dots), with means for each age group (black dots), and a linear regression (red line). (*B*) Their figure 4A showing similarity ratings for two example color pairs showing age-related “changes.” Data from individual participants are plotted (blue dots), along with means for each age group (black dots), and regressions (red lines). (*C*) Their figure 4B, showing for each color pair the regression beta between color similarity rating and age, with “significant” relationships indicated by asterisks. (*D*–*F*) Simulation of child results by adding age-dependent noise to an adult result for each simulated child participant. The observed patterns of results in panels (*A*–*C*) can be explained by greater noise in the ratings of younger participants.

We reanalyzed Moriguchi et al.’s data to simulate the performances of children by sampling the results of their adult participants and adding noise. For each simulated child, we randomly selected an adult and added two sources of age-dependent noise: i) a proportion of “random responses” for all color pairs except for those along the lead diagonal, varying between 20% for 3-y-olds and 5% for 12-y-olds; ii) additional variance to each rating, scaled inversely with age. Fig. 1 shows that noise can fully account for Moriguchi et al.’s findings for age-dependent changes in color similarity. This occurs because the 4-point rating scale constrains and skews the observed distribution of ratings. For example, for the red and blue pair in Fig. 1 *B* and *E*, greater noise for younger age groups increases mean ratings because the influence of noise at the lower end of the scale is truncated at 1, while noise at the upper end of the scale has a wider distribution. Moriguchi et al.’s results, therefore, provide no evidence for any age-dependent changes in mean underlying perceived color similarity.
